# Exploring the Combined Association between Road Traffic Noise and Air Quality Using QGIS

**DOI:** 10.3390/ijerph192417057

**Published:** 2022-12-19

**Authors:** Wisdom K. Adza, Andrew S. Hursthouse, Jan Miller, Daniel Boakye

**Affiliations:** 1 School of Computing, Engineering & Physical Sciences, University of the West of Scotland, Glasgow PA1 2BE, UK; 2 School of Health & Life Sciences, University of the West of Scotland, Glasgow PA1 2BE, UK

**Keywords:** environmental noise, air quality, environmental pollution, transportation, environmental public health, cardiovascular disease, Quantum Geographic Information System

## Abstract

There is mounting evidence that exposure to air pollution and noise from transportation are linked to the risk of hypertension. Most studies have only looked at relationships between single exposures. To examine links between combined exposure to road traffic, air pollution, and road noise. A Casella CEL-63x instrument was used to monitor traffic noise on a number of locations in residential streets in Glasgow, UK during peak traffic hours. The spatial numerical modelling capability of Quantum GIS (abbreviated QGIS) was used to analyse the combined association of noise and air pollution. Based on geospatial mapping, data on residential environmental exposure was added using annual average air pollutant concentrations from local air quality monitoring network, including particulate matter (PM_10_ and PM_2.5_), nitrogen dioxide (NO_2_), and road-traffic noise measurements at different component frequencies (Lden). The combined relationships between air pollution and traffic noise at different component frequencies were examined. Based on Moran I autocorrelation, geographically close values of a variable on a map typically have comparable values when there is a positive spatial autocorrelation. This means clustering on the map was influenced significantly by NO_2_, PM_10_ and PM_2.5_, and Lden at the majority of monitoring locations. Studies that only consider one of these two related exposures may exaggerate the impact of the individual exposure while underestimating the combined impact of the two environmental exposures.

## 1. Introduction

The process of urbanisation that drives increased noise and air pollution in the United Kingdom (UK) has consistently by 55.4% between 2007 and 2017 [1].

It is essential to understand the environmental hazard that noise and air concentrations have on community households [2,3]. Therefore, it is important to explore the combined exposure of high noise levels at different frequency components and air concentrations. This study quantitatively estimated the magnitude of noise pollution and air quality with health data to assess their risk to urban communities.

Noise and air pollution have detrimental health effects, as defined by World Health Organisation (WHO) [4,5,6]. The Sustainable Development Goals (SDG) goal 3 targets “ensuring healthy lives and promoting well-being for all ages.” Noise and air pollution are increasing globally, which has compelled several countries to develop regulations to govern them [7].

Road traffic noise studies have reported an association with auditory and more comprehensive health impacts such as hearing loss [8,9,10] and cardiovascular-related problems such as myocardial infarction and hypertension [11,12,13]. Annoyance and disturbed sleep are common effects because of road traffic exposure [14].

Observational studies indicate that significantly night-time noise increases levels of stress hormones and vascular oxidative stress, which may lead to endothelial dysfunction and arterial hypertension [14,15].

Some cohort studies in the UK have used the distance to major roads as a surrogate for exposure to air pollutants and traffic noise concerning cardiovascular health impact [16,17,18].

In the UK, a network of monitoring stations focuses on a range of six major air pollutants [19,20].

These pollutants include ozone, carbon monoxide (CO), nitrogen dioxide (NO_2_), sulphur dioxide (SO_3_), fine particulate matter (PM_2.5_), coarse particulate matter (PM_10_), and nitrogen dioxide (O_3_). To publish air quality levels that combine these pollutants, DEFRA utilises a specific UK air quality measure called the “Daily Air Quality Index” (DAQI). On a scale of 1 to 10, the DAQI rates air quality, with 1–3 representing “Low”, 4–6 denotes “Moderate”, 7–9 denotes “High”, and 10 denotes “Very High” [19,20,21,22].

Deteriorating air quality is a major problem in many nations. Significant pollution issues still challenge Scotland and the wider UK in general [23]. One of the first steps to a better understanding of the issue is the continuous monitoring of air quality.

The Scottish Government maintains a network of air quality monitoring sites in urban and rural locations making the information publicly accessible through the Scottish Air Quality Database [23]. 

Work to understand the effects of urban road traffic on air and noise pollution has been reviewed [24] and highlight the importance of other contributing factors. these such as dispersion models applied, relevant Geographic Information System (GIS)-based tools, spatial scale of exposure assessment, study location, sample size, nature of traffic data, and detailed building geometry. The most popular assessment method for short- and long-term exposure to noise and air pollution is deterministic modelling. Compared to applied noise models, it was found that air pollution models had a wider variety The correlations between air and noise pollution vary widely (0.05–0.74) and depend on several factors, including traffic and building characteristics, and meteorological conditions [24].

Buildings serve as filters for the spread of pollutants, although the influence on noise pollution is far greater than that of air pollution. While also crucial for noise pollution, meteorology has a higher impact on air pollution levels than it does on noise. To aid in the facilitation of studies pertaining to health, there is a substantial potential for the development of a standard method to evaluate combined exposure to traffic-related air and noise pollution. The use of GIS is well established and has a significant capacity to simultaneously handle both exposures because of its spatial character [24].

Due to the problems that noise, and air pollution pose, finding the best approach to control them is essential. One of the most challenging procedures in existence is managed by urban planners and public health specialists [25]. It involves controlling vehicle traffic noise and air pollution [25]. Additionally, a study conducted in Shirdi, India, found that noise levels and air pollution exceeded the recommended limit. The authors suggested that the government should put action plans in place, such as facilitating proper management of traffic signals. Secondly, the government should divert all traffic to another subway. Thirdly, the government should declare Shirdi city as “No Horn city”. Fourthly, the government should implement measures to control pollutant emissions. Finally, they suggested that government should conduct a long-term study on concentrations of particulate matter in the spatial and temporal dimensions of various cities due to the development of infrastructure [26].

The joint relationship between road traffic noise at various frequencies and air pollution, however, is not well understood [11]. By analysing combined associations between modelled noise levels at various frequency components and air concentration utilising other spatial units and area attributes, this study explores this using GIS models [27,28]. 

The empirical evidence focuses on information from road traffic noise pollution and air concentration gathered by local observations.

In order to identify locations where exposure to noise levels and air pollutants that are greater than the limit values, noise pollution and air quality were mapped using QGIS software. As a result, one of the objectives of this study was to ascertain the annual average residential exposure to air (μg/m^3^) and noise pollution (Lden), at five different locations in urban Glasgow.

The noise levels were assessed using two different techniques. The actual measurements of traffic noise were taken using the Casella CEL-63x equipment. Noise pollution simulation was carried out using QGIS software and its specialised plugin opeNoise [25]. The opeNoise plug in is one of many QGIS options and allows users to compute the noise level produced by a point source or a road source at fixed receiver spots and structures. IDW (Inverse Distance Weighted) interpolation for a point vector layer was another processing tool used to create raster layers and structures that are central to receiver locations and interpolate the air quality index at a site from a point source or a road source using improved IDW. Then, map the NO_2_, PM_10_, and PM_2.5_ concentration monitoring data. using the Air quality in Scotland website.

The mapping of noise and air quality enables the identification of actions that should be taken to lower noise levels and air pollutants to safeguard public health. This study used noise and air quality mapping to better understand how traffic air and noise pollution vary in space and their combined association.

The goal is to create an informative spatial visualisation from this data and joint association to evaluate potential links to health outcome indicators.

## 2. Materials and Methods

**Figure 1:**
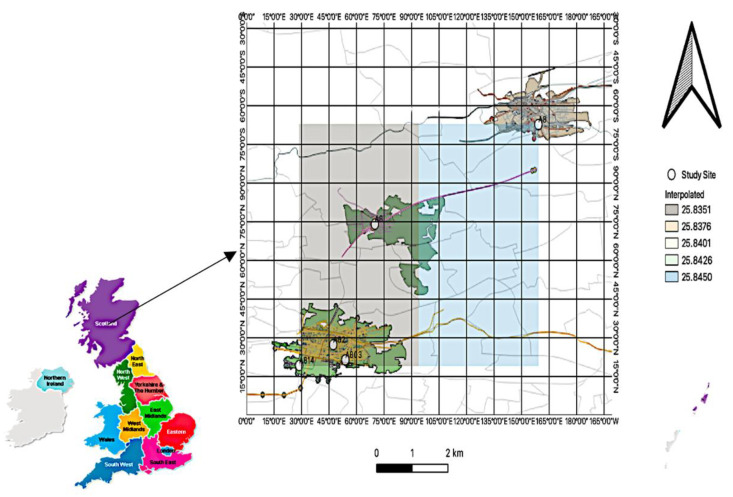
Map of study area. The maps were created using the QGIS 3.24.3-Tisler software (Open Source Geospatial Foundation Project, Chicago, IL, USA).

This study was conducted at roadside locations in residential areas of Greater Glasgow. Five study areas (Glasgow High Street, Glasgow Towhead, East Dunbartonshire Bishopbriggs, East Dunbartonshire Kirkintilloch, and Glasgow kerbside) were used for data collection (Figure 1). Located in the largest urban area in the central belt of Scotland, they are characterised by heavy traffic on major roads with urban residential settings. Population density and nearest neighbour in various communities were considered for exposure assessment.

**Figure 2:**
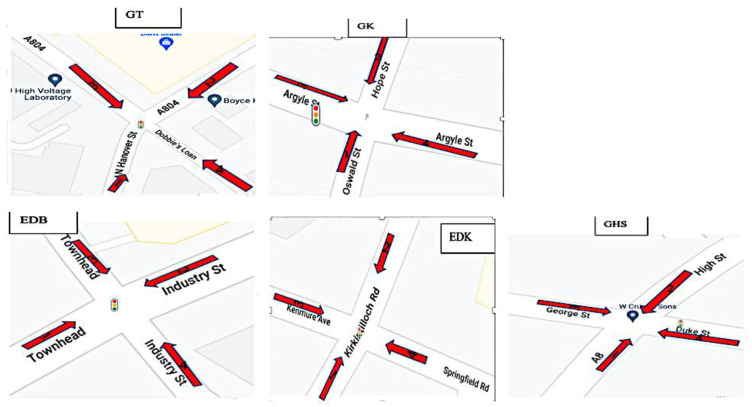
Directions of traffic flow at the monitoring locations.

**Figure 3:**
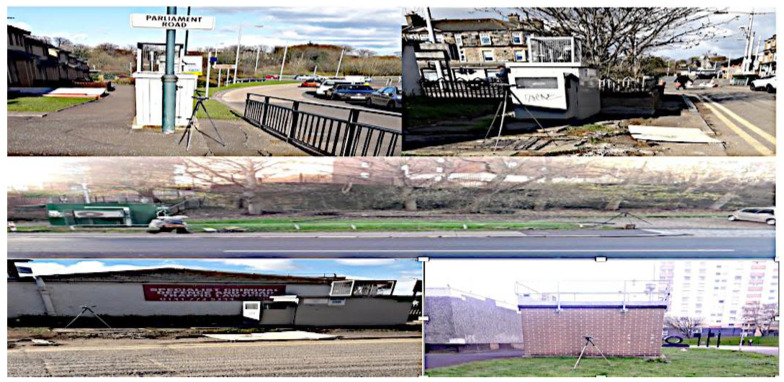
Position of sound meter and air monitor at monitoring sites.

Monitoring locations used for noise measurement included five major urban traffic road junctions close to around 500 residential areas. These locations were selected using feedback from the air quality monitoring data and major-traffic road sites (with perceived high-traffic noise and air pollution data) based on urban background criteria [22]. To locate monitoring locations for air pollutants as efficiently as possible, noise sampling sites were chosen using a location-allocation model. The study incorporated land use, road networks, population density, and regulatory air-quality monitoring. The study focused on the directions of traffic flow at the monitoring sites (Figure 2 and Figure 3). These places are;

EDK—Four-way intersection of the street leading to four ways round about: Townhead and Industrial Street,

GT—Four-way intersection of the street: St. Mungo Avenue, Kennedy Street, North Hanover Street, and Cowcaddens Road

GK—Four-way intersection of the street: Hope Street, Argyle Street, and Oswald Street

EDB—Four-way intersection of the street: Kenmure avenue, Bishopbriggs Cross, Kirkintilloch Road, and A803.

GHS—Four-way intersection of the street: Duke Street, George Street, castle street, and A8.

In addition to high road traffic, these locations contain residential buildings with high housing density, business facilities, scientific and educational institutions, health, and other public institutions, such as the administrative buildings of the local authority.

Figure 2 depicts five approaches to crossroads, each with arrows and numbers indicating possible traffic flow routes through the crossing.

The amount of traffic noise emitted depends on factors such as traffic load. As a result, in addition to the criteria indicated in (Table 1), when estimating noise levels in metropolitan areas, one of the most important aspects of road traffic is the load on the road or traffic flow. The number of cars passing through the intersection’s centre in a unit of time is the most straightforward way to define traffic flow.

Counting traffic on the roads was automatic, meaning the number of cars passing through a specific road segment in a certain time frame was estimated using the previous year’s Annual Average Daily Flow (AADF) method. The direction of movement and the type of vehicle used automatic basic counting patterns to record the counting when the cars passed. Automatic counts were compared with the manual count for accuracy in traffic flow counts. This study considered the estimation using the previous year’s Annual Average Daily Flow (AADF) method in the five specific study locations. An AADF is the daily average number of vehicles passing a site on the road network over a year.

The study considers the Road Traffic Act 1930 on the UK’s classification of a motor vehicle. The following are the vehicle categories used in this study:

Two-wheeled motor vehicles, M—passenger-carrying vehicles, abbr. PCVs (Car and taxi vehicles), N—goods-carrying vehicles (lorries and vans)—trailers and semitrailers, Light-goods vehicles (lgvs), Buses and coaches, and all heavy goods vehicles (all hgvs).

Because the noise emission of different vehicle categories varies substantially, these categories are studied. On the other hand, the two-wheeled type of vehicle is included in our research since it impacts overall traffic noise. The interaction of the wheels with the road surface and the airflow demonstrates this influence.

Data was taken on the annual daily road traffic flow of Car and taxi vehicles, Two-wheeled motor vehicles, Light-goods vehicles (lgvs), Buses and coaches, and all heavy goods vehicles (all hgvs) at each of the sites (Glasgow High Street, Glasgow Townhead, East Dunbartonshire Bishopbriggs, East Dunbartonshire Kirkintilloch, and Glasgow kerbside) during the monitoring period from the department of transportation (road traffic statistics) on road traffic flow [29].

The population affected, traffic flow, and high risk exposed individuals were also considered in the outcome of this study.

### 2.1. Data Collection Techniques

Measurements of noise and air quality were made simultaneously across (Glasgow High Street, Glasgow Townhead, East Dunbartonshire Bishopbriggs, East Dunbartonshire Kirkintilloch, and Glasgow kerbside). Information was collected on traffic flow rates, road width in front of each site, and meteorological factors from the noise Scotland website and air quality network database, specifically in Glasgow [22,29]. Estimated Background Air Pollution Maps (2018) [22] at total annual mean concentrations based on the grid squares of 1 km × 1 km [10] were downloaded. This data from DEFRA consists of several coordinates in all regions of Scotland. This study used the annual daily average air concentration (NO_2_, PM_10_ and PM_2.5_) from five locations in the central belt of Scotland from 23 November 2020, to 24 November 2021, for this modelling. Data were obtained from the Openair LAQM Data Download Site [22]. The air pollution data was collected from the Scottish air quality monitoring network and only limited to urban city areas. Air quality is monitored under the operating principles of automatic analysers using tapered element oscillating microbalance (TEOM) equipment [30]. The monitoring was completed using automatic analysers reference techniques of measurement, except for the automatic PM_10_/PM_2.5_ analysers, which are those that are outlined in the EU Directives. The standard procedure for measuring NO_2_, PM_10_ and PM_2.5_ levels was completed using chemiluminescence, flow dynamic measurement system (FDMS), beta attenuation monitor (BAM), and gravimetric sampler respectively [30].

### 2.2. Noise Level Monitoring

During the field noise monitoring, the Casella CEL-63x and 1/3 Octave Band Sound Level Meter were sited at 1.2–1.5 m on a tripod, at 1m from the road edge adjacent to the air quality monitoring equipment. The instruments were often at least 10 m from the centre line of the nearest traffic lane in Glasgow (depending on the road type). It was at least 1 m away from buildings to avoid reflection from each site location and a consistent and safe distance from the road. It is kept within sight and supervised by the researcher 100% of measurement time for equipment security and to reduce any issues should the public or workers require access to the location. Hence, the noise measurements were conducted in the same place government sited air quality monitoring equipment (TEOM).

The daily community noise exposure level was measured for 3 to 6 days during the daytime in the morning, afternoon, and night as Lden and Lnight, respectively. The road traffic noise was measured at the height of 1.2–1.5 m on a tripod, at 1m away from the road edge adjacent to the air quality monitoring equipment. The environmental conditions involved dry road surfaces, no rain, a humidity of 5 % to 90 % RH non-condensing, a temperature of 10 °C to +50 °C (Class 1), 0 °C to +40 °C (Class 2), pressure of 65 kPa to 108 kPa and a wind speed below five m/s.

The measurement times were taken at a minimum of 1 h to 4 h in length most often at the end of a daily measurement.

The community noise measurement and air quality monitoring start during the daytime between (09:00–17:00).

The community noise measurement processes and the setup of noise monitoring at various sites followed the UK Calculation of Road Traffic Noise (CRTN) method and CNOSSOS-EU standard method of exposure noise mapping for major roads considering agglomeration to check environmental noise standards [31,32]. The study defines community noise calculations in the range of 16Hz to 16 kHz, and frequency band results were provided at the corresponding frequency interval. An analysis was performed in octave bands for road traffic [8]. For road traffic noise, based on these octave band results, the A-weighted long-term average sound pressure level for the day, evening, and night period, as defined in Annex I and referred to in Art. 5 of Directive 2002/49/EC, is computed by summation over all frequencies:

LAeq, T = 10 × lg∑_(i = 1) 10^((Leq, T, i ± Ai)/10)(1)

where Ai denotes the A-weighting correction according to IEC 61672-1

i = frequency band index and

T is the period corresponding to daytime, evening, or night.

Simulation of road traffic noise pollution

QGIS 3.10.2 simulated road traffic noise pollution in the Greater Glasgow territory. This software is a cross-platform desktop geographic information system application that facilitates viewing, editing, and analysing geographical data. It is free and open source.

As in [25], the simulation of road traffic noise measurements was modelled using QGIS.

The importing of the shapefile map of Scotland into a suitable coordinate system is the first step in simulating road traffic noise pollution. EPSG:27700-OSGB36/British National Grid– Projected is the coordinate reference system in this situation.

Building two essential layers [25] to represent the ambient noise caused by vehicle activity in Greater Glasgow is the second step: Buildings and Roads.

The initial layer defines the polygonal buildings and the design of structures using the existing blueprints.

Buildings were labelled with different colours based on the amount of noise they were exposed to. The maximum noise level of residential facilities located at the intersection was recorded. The noise level from road was assigned to building.

According to the UK’s regulatory standards, noise on the facade of evaluated facilities should not exceed 65 dB(A).

The structures were drawn using a shapefile map of Greater Glasgow already entered. The building’s purpose was not considered during the design. The second stratum was identified as the roads. This layer indicates the source of traffic noise emission, and the road layer’s type is a line type.

The noise level at reception sites caused by road sources (a line layer) was determined.

Simulations were run for various periods, more specifically for data collected during the daytime at 14 h, evening time at 2 h at the penalty of +5, and night-time at 8 h at the detriment of +10 by Directive 2002/49/CE and UK Calculation of Road Traffic Noise (CRTN) method and CNOSSOS-EU standard method. The plugin automatically calculates the value of Lden when data referred to the three reference periods are set (Daytime, Evening time, and Night-time).

The research ray is the maximum distance of influence of the source to the receiver in meters—receivers’ points beyond the research ray return a −99 value. A smaller research ray reduces the calculation time with consequent loss of precision in sound-level estimates. The research ray was set to 1000 m. Therefore, the relative humidity to 70 and the air temperature to 20 degrees Celsius by the ISO 9613–1.

The following additions were made; data on the annual daily road traffic flow of Car and taxi vehicles, Two-wheeled motor vehicles, Light-goods vehicles (lgvs), Buses and coaches, and all heavy goods vehicles (all hgvs) at each of the sites during the monitoring period from the department of transportation (road traffic statistics) [29].

### 2.3. Modelling of Road Traffic Air Quality

Since the same location from the simulation of road traffic noise was used, the OpenStreetMap (OSM) and shapefile will clearly show the point and location. However, the noise level was represented by points, so it will be appropriate to have air quality as a raster layer. Inverse density weight (IDW) was applied to air pollutants to build spatial weight matrices for spatial autocorrelation studies.

Firstly, the interpolation attribute is selected and the actual pollutant data of NO_2_, PM_10_, and PM_2.5_ was inserted at the original coordinate of X and Y (Latitude and Longitude). The interpolation was run, and the raster layer produced by the interpolation was edited. The editing was made by opening the Layer Styling Panel by clicking it. Select and change the single band grey to single band pseudocolour renderer from the drop-down menu. Select Cumulative count cut from the Min/Max Value Settings section. Choose a spectral colour ramp. The spectral colour was inverted to match the pollutant and renamed the contaminant level according to the national air quality index to interpret the air pollutant. After applying the style, the air quality index in legend form, among other layers could be seen. The air quality index was layered according to the Committee on the Medical Effects of Air Pollutants [19,20,21].

This work used the hourly mean concentration for each air pollutant including nitrogen dioxide. The latest 24-h running mean for the current day is based on the daily mean concentration for secondary data for PM_2.5_ particles. The present day’s 24-h running mean is calculated using the daily mean concentration for secondary data for PM_10_ particles. This study used the annual daily average air concentration (NO_2_, PM_10_ and PM_2.5_) from 23 November 2020 to 24 November 2021 for this modelling.

This research performed all statistical analysis using R analysis, Python Consoles and QGIS 3.24.3-Tisler. This research conducted spatial analysis on joint road traffic noise and air pollutants to model data into several shapefiles’ maps. This map consists of a vector layer with attributes information raster layer. The geospatial analysis in geometric location includes the data of noise, air pollutants, residential buildings, and road traffic flow rate for each monitoring site in the central belt of Scotland.

The Moran I was used to analyse spatial autocorrelation of joint noise level and air pollutants (Lden, NO_2_, PM_10_ and PM_2.5_) considering the number of simulations equal to 100 and the number of neighbours (nearest) to be 10 to 100 km.

Inverse density weighting (IDW) interpolation of NO_2_, PM_10_ and PM_2.5_ were aggregated.

An analysis of noise level (Lden) at road traffic and residential buildings during the daytime, night, and evening was simulated and analysed at monitoring sites.

## 3. Results

### Geospatial Results of Data

Data results from an air quality index (AQI) description and noise levels in a spatial view were joined. The data contains the number of air pollutants in each particulate matter less than 10 microns (PM_10_), nitrogen dioxide (NO_2_), particulate matter less than 2.5 microns (PM_2.5_), and noise level in Lden, which was computed from daytime, night-time, and evening time noise level dB(A).

**Figure 4:**
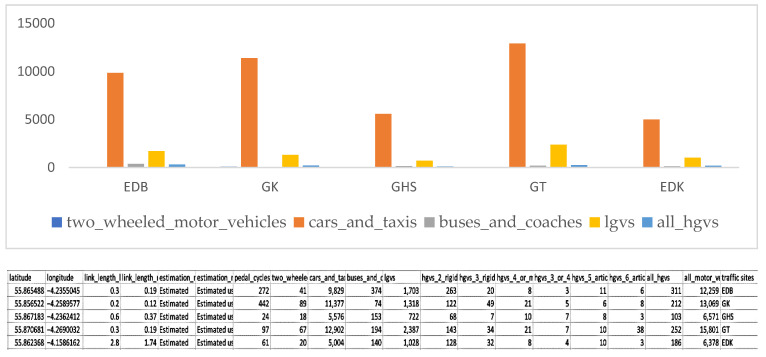
Annual daily traffic flow of the type of vehicles in various monitoring sites.

Figure 4 above depicts the difference in the monitoring sites concerning the annual daily traffic flow. The maximum and the minimum number of traffic flows were 12,902 (cars and taxis) at GT and 20 (two-wheel motor) at GHS, respectively. Car and taxi vehicles in all the monitoring sites have the highest traffic flow, from 5004 to 12,902 vehicles. Afterwards, low goods vehicles (lgvs) similarly had the following highest number of traffic flow in all the monitoring sites, from 722 to 2387 vehicles. The two-wheel motor had the minimum annual traffic flow at all the monitoring sites, from 18 to 89 vehicles. The findings clearly show a high daily traffic flow at all the monitoring sites.

From Table 2 above, the Car and taxi, lgvs, and all hgvs traffic flow rate was significantly (all *p*-values 0.050) linked with the low-frequency components (31.5 Hz–125 Hz) at 63 Hz and 125 Hz band of traffic noise and high-frequency traffic noise components between 1 KHz to 8 KHz band and with strong correlation coefficients (rho ranged from 0.900 to 1.000).

All hgvs had high-frequency traffic noise components at 8KHz substantially (all *p* values 0.050) but strongly correlated (rho = 0.900). There was a positive correlation across the frequency range. Only the buses were significant with PM_10_ and had a negative strong correlation co-efficient rho = −0.894.

The total traffic flow rate was significant (all *p*-values 0.050) although strongly correlated (rho ranged from 0.900 to 1.000) with most high and low-frequency traffic noise components.

From Table 3, it was observed that the correlation between PM_10_ and NO_2_ spearman’s rho (R) value of 0.380 was statistically significant since the results showed a smaller *p*-value of 0.001. The correlation between (PM_2.5_ and NO_2_) and (PM_2.5_ and PM_10_) (spearman’s rho value 0.345) and 0.897 respectively was statistically significant since results showed a smaller *p*-value of 0.001.

Table 3 also shows that the relationship between the mid-frequency component and PM_2.5_ spearman’s rho value 0.145 was statistically significant because their *p*-values were lesser than the significance level of 5%. On the other hand, the relationship between mid and low noise frequency component spearman’s rho value of 0.852 was statistically significant since results showed a smaller *p*-value of 0.001. Additionally, the relationship between high, and low noise frequency component spearman’s rho value of 0.864 was statistically significant due to a smaller *p*-value of 0.001. Similarly, the relationship between high, and mid noise frequency component spearman’s rho value 0.976 was statistically significant since results showed a smaller *p*-value of 0.001. The correlation is high and significant since there was a smaller *p*-value of 0. 001 across air concentration and noise at different frequency components.

This implies that the risk of hypertension associated with (mid noise frequency component and PM_2.5_), (PM_10_ and NO_2_), (PM_2.5_ and NO_2_), (PM_2.5_ and PM_10_), (mid and low), (high and low), (high and mid noise frequency component) respectively.

The simulation of annual road traffic noise pollution in Greater Glasgow is in five different locations (23 November 2020–23 November 2021).

It carried out a comparison of measured and modelled data. In that situation, it might be determined that there are various buildings affected by road traffic noise. The simulation enables traffic noise to be assigned to facilities that enhance the clarity of how noise affects residents and be recommended for noise assessment and planning.

**Figure 5:**
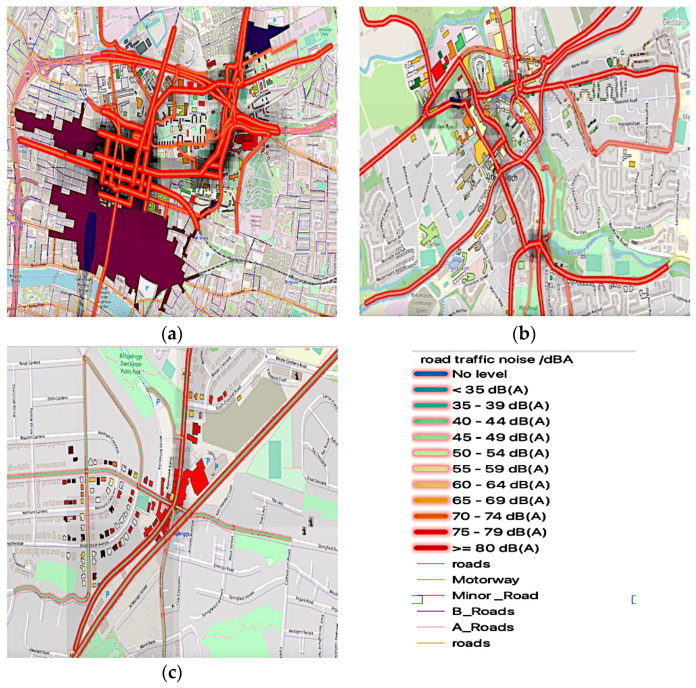
A model of road traffic annual average noise level (Lden) at monitoring areas at five monitoring locations (23 November 2021): (**a**) road traffic noise (Lden) at GHS, GK, and GT; (**b**) road traffic noise (Lden)at EDK; (**c**) road traffic noise (Lden) at EDB.

Buildings are labelled with different colours based on the amount of noise produced by the road. Maximum noise level is recorded at the various locations of the facades of buildings located at the intersection and areas along the route. Our findings show that the annual (Lden) noise level of some of these structures is at the highest level at GK, GHS, and EDB, exceeding 80 dB(A), as shown in Figure 5a,c. However, as shown in Figure 5b, the EDK max level was between 65 to 69 dB(A), and during peak hours, buildings near crossings are subject to noise levels exceeding 80 dB(A). This finding concludes that the residents in the areas are loud, and noise on the facade of evaluated facilities should not exceed 65 dB(A) according to Scotland’s regulatory standards. Due to their location along the city’s busiest roadways, the analysed buildings are the most endangered by noise.

**Figure 6:**
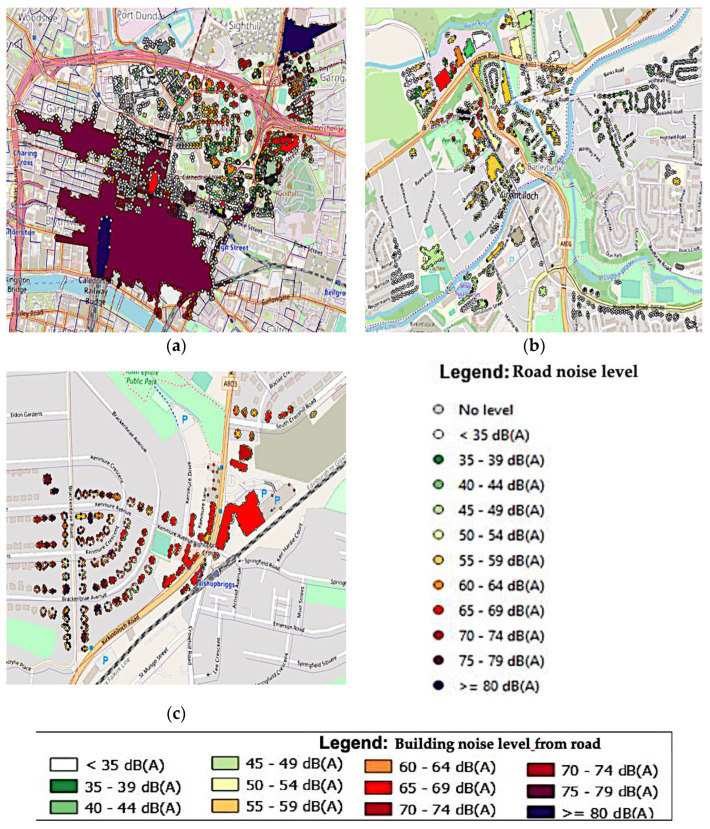
A model of annual road traffic noise levels (Lden) at monitoring areas at five monitoring locations (23 November 2021): (**a**) Community noise level (Lden)at GHS, GT, and GK; (**b**) Community noise level (Lden)at EDK; (**c**) Community noise level (Lden) at EDB.

Figure 6 depicts the structure at a specific site in the Lden (day, evening, and night). The noise levels at the receiver points on one system are noticeably different. For example, in the building depicted in Figure 6a–c, there are two receiver locations with noise levels greater than 80 dB(A), one receiver point with annual noise levels of 75–79 dB(A), and one receiver point with noise levels of 65–69 dB(A), and one receiver point with an annual noise level of 50–54 dB(A). It was observed in Figure 6c that, the EDB area shows a high noise level. Therefore, EDK (b) is the second most endangered building.

#### Joint Annual Average of Air Pollutants (μg/m^3^) and Noise Pollution at Lden

**Figure 7:**
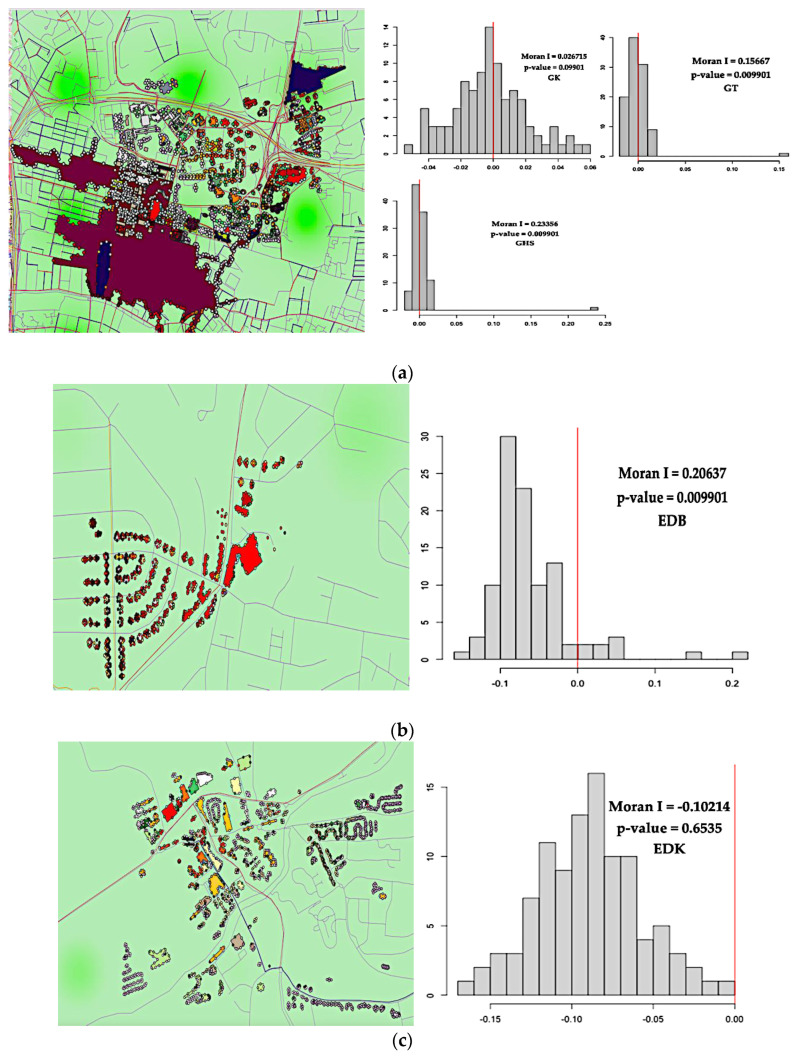

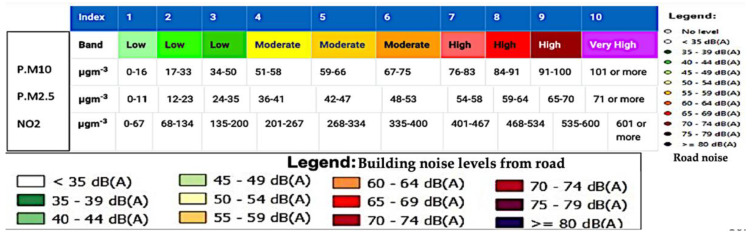
Joint annual average NO_2_, PM_10_ and PM_2.5_ concentration(μg/m^3^), and noise level (Lden) at five monitoring locations (23 November,2021): (**a**) Joint air-noise pollution (Lden)/(μg/m^3^) at GHS, GT and GK; (**b**) Joint air-noise pollution (Lden)/(μg/m^3^) at EDB; (**c**) Joint air-noise pollution (Lden)/(μg/m^3^) at EDK.

As shown in Figure 7a–c, information depicts air and noise pollution levels, and actionable recommendations and health advice can also be deduced. The air quality index number is 1 and 2, showing 0–16 (μg/m^3^) and 17–33 (μg/m^3^) annual average PM_10_ concentration regarding air pollution levels at all five monitoring areas, respectively. The air quality findings mean individuals in that areas can enjoy their usual outdoor activities with low risk. However, as previously discussed, noise levels exceed the limit for individuals’ exposure in the buildings. The residents at EDB, GHS, and GK live in buildings with high noise exposure.

Figure 7a shows joint air and noise pollution levels. The air quality index number is one, and two offer leading (band). The annual average PM_2.5_ concentration (μg/m^3^) regarding air pollution levels at all five monitoring areas was between 0–11 except in three monitoring areas (GHS, GT, and GK) Figure 7b, annual average PM_2.5_ concentration was 12–23 (μg/m^3^) respectively. According to actionable recommendations and health advice, it is recommended that individuals in that areas can enjoy their usual outdoor activities with no risk. Coincidentally, Figure 7a,b shows, noise levels exceeding 65 dB(A), especially among EDB, GHS, and GK residents. There are unsafe levels of road traffic noise, making individuals in the building highly endangered and prone to the risk of hypertension.

Figure 7a,b shows joint air and noise pollution levels. The air quality index number is 1, 2, 3 leading low (band). The annual average NO_2_ concentration regarding air pollution levels at all five monitoring areas was between 0 and 159 (μg/m^3^), respectively. According to actionable recommendations and health advice, it is recommended individuals in that areas can enjoy their usual outdoor activities with low risk. Coincidentally, noise levels exceeding 65 dB(A), especially among residents in the EDB, GHS, and GK. There are unsafe levels of road traffic noise, with individuals in the buildings at risk of increased hypertension.

As shown in Figure 7b, the model was statistically significant because their *p*-values were lower than the significance level of 5%, and the *p*-value was 0.009901. The findings indicate that there was clustering in the map. If autocorrelation exists in a map, the condition that observations are independent of one another is violated. The plot shows the distribution of Moran’s I values; the red vertical line shows our observed Moran’s I value of 0.20637. The *p*-value provided by the Monte-Carlo (MC) simulation was 0.01, indicating a 1% risk of rejecting the null hypothesis incorrectly or a 1% chance that our observed pattern is consistent with a random process. There is a similar observation of values from road traffic noise (Lden) and NO_2_, PM_10_, and PM_2.5_ at the EDB monitoring area. There was a positive spatial autocorrelation association between noise levels (Lden) and air pollution concentrations (NO_2_, PM_10_ and PM_2.5_).

On the other hand, as shown in Figure 7c, there was a negative spatial autocorrelation but not statistically significant because their *p*-values were not lesser than the significance level of 5%. The *p*-value is 0.6535, indicating there was dispersion in the map. The plot shows the distribution of Moran’s I values, and the red vertical line shows our observed Moran’s I value of −0.10214. The *p*-value provided by the MC simulation was 0.65, indicating a less than 1% risk of rejecting the null hypothesis incorrectly or a less than 1% chance that our observed pattern is consistent with a random process. Therefore, road traffic noise (Lden) is not influenced by NO_2_, PM_10_ and PM_2.5_ at the EDK monitoring locations.

From Figure 7a it was observed that the model was statistically significant because their *p*-values were lower than the significance level of 0.1%. The *p*-value is 0.09901, which indicates that there was clustering in the map. The *p*-value provided by the MC simulation was 0.1 and our observed Moran’s I value of 0.0267, indicating a 1% risk of rejecting the null hypothesis incorrectly or a 1% chance that our observed pattern is consistent with a random process. There was a positive spatial autocorrelation association between noise levels (Lden) and air pollution concentrations (NO_2_, PM_10_ and PM_2.5_).

Road traffic noise (Lden) is associated positively with NO_2_, PM_10_ and PM_2.5_ at the GK monitoring locations.

From Figure 7a, it was observed that, the model was statistically significant because their *p*-values were lower than the significance level of 5%, and the *p*-value was 0.009901. Findings indicate that there was clustering in the map. The plot shows the distribution of Moran’s I values; the red vertical line shows our observed Moran’s I value of 0.15667. The *p*-value provided by the MC simulation was 0.01, indicating a 1% risk of rejecting the null hypothesis incorrectly or a 1% chance that our observed pattern is consistent with a random process. There is a similar observation of values from road traffic noise (Lden) and NO_2_, PM_10_ and PM_2.5_ at the GT monitoring area. There was a positive spatial autocorrelation association between noise levels (Lden) and air pollution concentrations (NO_2_, PM_10_ and PM_2.5_). That is, road traffic noise (Lden) is being influenced by NO_2_, PM_10_ and PM_2.5_ at the GT monitoring area significantly.

## 4. Discussion

The noise measurements at locations in Greater Glasgow suggest that communal noise levels are high. Noise levels exceed the specified values for the areas of purpose—the city centre, craftsman, commercial, and administrative areas with housing, zones along highways, main roads, and city traffic arteries, which are 65 dB(A) for day/evening—in practically every measuring point. In the afternoon, the equivalent noise level at locations GHS, EDK and GK reach a values of 86.8 dB(A) to 87 dB(A). A noise pollution simulation was developed using the findings of field noise measurements and a study of the traffic regime, i.e., traffic load at the observed locations. The simulation was complete using the opeNoise plugin and IDW and R analysis in QGIS software. After the simulation, receiver sites on half of each building’s facade in the vicinity of the intersection were calculated, as well as the noise intensity and interpolation of air pollutants

Each structure was given a noise level. The maximum value among all the receiver points for that building is the value assigned to it.

The annual road traffic noise in Greater Glasgow in five different locations were exhibited. According to the noise assessment, the noise given to the building is the most significant value among all the noises. The simulation shows that the maximum and annual average noise level in a number of buildings in GK and GHS, EDB is more than 80 dB(A) because the noise assigned to the building is the highest value among all the building receiver locations. The air quality index number is 1,2,3 showing a low (band). The annual average NO_2_, PM_10_ and PM_2.5_ concentrations regarding air pollution levels at all five monitoring areas were between 0–35, 0–50, and 0–200 (μg/m^3^), respectively. According to actionable recommendations and health advice on the low band, it recommended individuals in that area can enjoy their usual outdoor activities with low risk. This form of simulation is not always appropriate because it implies that all building residents are subjected to the same maximum levels of traffic noise and air pollution, which is not always the case. To reduce these variations and anomalies, the noise level and air concentration measurements were assessed in numerous distinct areas at specific locations in the study to ensure that the simulation is valid. This study was conducted within the COVID-19 pandemic period, examining all environmental exposures between 2020 and 2021.

Both noise and air pollution showed different spatial patterns, and correlations across monitoring areas varied substantially depending on the unit of analysis: Greater Glasgow, neighbourhoods, and 1 km × 1 km grid cells. Auto spatial correlations across all monitoring areas in Greater Glasgow were moderately strong. They varied depending on the exposure metrics between NO_2_, PM_10_ and PM_2.5_, and Lden of Moran I value 0.4136 is closest to 1. Auto spatial correlations were mostly stable across exposure terrains, distance to significant road intersections, and deprivation terrains. The plot shows the distribution of Moran’s I values, and the red vertical line shows our observed Moran’s I value of 0.4136. The *p*-value provided by the MC simulation was 0.01, indicate is a 1% risk of rejecting the null hypothesis incorrectly or a 1% chance that our observed pattern is consistent with a random process. There is a positive spatial autocorrection because the Moran I value of 0.4136 is closest to 1.

A similar study illustrated how to use a spatial unit and local characteristics to analyse the relationship between air pollution and road traffic noise in London between 2003 and 2010 [11] but slightly different findings.

From our study we can conclude: 

Noise monitoring in the selected communities indicated road traffic generated high noise levels above safe levels recommended by OSHA, WHO, Environmental Protection (Act) 1990, Noise and Statutory Nuisance Act of 1993, and community noise regulatory bodies.

The noise exposure of road traffic performances exceeded recommended levels in some communities such as Glasgow High Street, East Dunbartonshire Bishopbriggs, and Glasgow kerbside.

Even though the air pollution level was low, some exceeded recommended level [4,30]. Annual average PM_2.5_ concentrations should not exceed 5 g/m^3^, and 24-h average exposures should not exceed 15 g/m^3^ more than 3–4 days per year, according to the new guidelines.

Interim targets have been established to aid in planning incremental milestones towards cleaner air, particularly for cities, regions, and countries with significant levels of air pollution. In the case of PM_2.5_, they are an annual mean of 35 g/m^3^, a 24-h standard of 75 g/m^3^, a yearly mean of 25 g/m^3^, a 24-h mean of 50 g/m^3^, an annual mean of 15 g/m^3^, 24-h mean of 37.5 g/m^3^ and yearly mean of 10 g/m^3^, 24-h mean of 25 g/m^3^.

The PM_10_ concentrations 15 g/m^3^ every year and 45 g/m^3^ on a 24-h basis. NO_2_ concentrations of 10 g/m^3^ every year and 25 g/m^3^ on a 24-h basis.

When considering the current policy and air quality index, WHO and EU air quality standards were appropriate [30].

From our findings, residents at a number of locations may subject to excessive noise and air pollution. Prolonged exposure may have long-term and short-term health repercussions such as physiological stress responses. Most temporary and short-lived symptoms include hypertension, digestive and immune system illnesses, impaired concentration and memory, and a restricted visual field. They can also become chronic (insomnia, high blood pressure, anxiety, depression). Therefore, it was necessary to look at the risk of hypertension.

Our demonstrates the joint association of air concentration and frequency components of road traffic noise. According to the findings, those who live in areas with heavy traffic noise at (mid and high frequencies of 250, 500, 1000, 2000, 4000, and 8000 Hz) and high concentrations of PM_10_ μg/m^3^ are much more likely to develop hypertension.

The results at 63 and 125 Hz are consistent with the idea that exposure to low-frequency (10–200 Hz) traffic noise may cause hypertension by irritating cortical or subcortical areas via the neuroendocrine system [11].

Additionally, there was a strong correlation between exposure to low-frequency noise and Car and taxi, light goods vehicles traffic. There was a strong correlation between exposure to mid-frequency noise and light goods vehicle traffic. Additionally, there was a strong correlation between air concentration exposure to PM_10_ and bus traffic. Motorcycles were the main cause of traffic noise and air pollutants in Greater Glasgow because they had the highest correlation coefficients, with noise levels at 63 Hz, 125 Hz, 1000 Hz, 2000 Hz, 4000 Hz, and 8000 Hz.

Due to their notable hearing sensitivity at roughly 1000 Hz, people with normal hearing may be one explanation for the greater risk seen at this frequency [9]. Another possibility is that isolated auditory brainstem responses to noise exposure of more than 30 dB at 1000 Hz were found to be 100% in young adult research [8]. However, evidence of a significant correlation was found at PM_10_ and NO_2_.

The strong association between noise exposure and total traffic flow rate, is not unexpected. The total traffic flow rate was significant (all *p*-values 0.050) although strongly correlated (rho ranged from 0.900 to 1.000) with mostly high and low-frequency components of traffic noise.

The correlation between PM_10_ and NO_2_ spearman’s rho (R) value of 0.380 was statistically significant with a lower *p*-value of 0.001. The correlation between (PM_2.5_ and NO_2_) and (PM_2.5_ and PM_10_) (spearman’s rho value 0.345) and 0.897 respectively were also statistically significant (*p*-value of 0.001).

The relationship between the mid-frequency component and PM_2.5_ (spearman’s rho value 0.145) was statistically significant because their *p*-values were lower than the significance level of 5%. On the other hand, the relationship between the mid and low noise frequency component (spearman’s rho value 0.852) was statistically significant due to a smaller *p*-value of 0.001. Additionally, the relationship between high and low noise frequency components (spearman’s rho value 0.864) was statistically significant because of a smaller *p*-value of 0.001. Similarly, the relationship between high, and mid noise frequency components (spearman’s rho value 0.976) was statistically significant because of a smaller *p*-value of 0.001. The correlation is high and significant due to a smaller *p*-value of 0. 001 across air concentration and noise at different frequency components.

This may lead to hypertension by irritating cortical or subcortical areas via the neuroendocrine system. Hypertension may be influenced by combined (mid noise frequency component and PM_2.5_), (PM_10_ and NO_2_), (PM_2.5_ and NO_2_), (PM_2.5_ and PM_10_), (mid and low noise frequency component), (high and low noise frequency component) and (high and mid noise frequency component) respectively.

A similar study has been conducted in Taichung, Taiwan [33]. Results showed that the annual average LAeq, 24 h in Taichung was 66.4 ± 4.7 dBA. Significant differences in LAeq, 24 h, and frequency components were observed between land-use types (all *p*-values < 0.001), but not between seasons, with the highest two noise levels of 71.2 ± 1.0 dBA and 70.0 ± 2.6 dBA measured in stream-channel and areas, with the highest component being 61.4 ± 5.3 dBA at 1000 Hz. Road width, traffic flow rates, and land-use types were significantly associated with annual average LAeq, 24 h (all *p*-values < 0.050). Noise levels at 125 Hz had the highest correlation with total traffic (Spearman’s coefficient = 0.795) and the highest prediction in the multiple linear regression (R² = 0.803; adjusted R² = 0.765). The study reported noise levels higher than the permissible levels of 80 dB.

It is limiting to combine the effects of noise exposure with air pollution exposure in this study because of the substantial correlation.

Additionally, it was more effective to monitor various frequency components of road traffic noise and air concentration to demonstrate the relationship between combined exposures.

The noise measures only consider a person’s immediate exposure to road traffic noise and extrapolates to their long-term exposure. A 1 h LAeq at the five sampling sites is likely to be a reliable indicator of long-term noise levels [33].

## 5. Conclusions

This study has demonstrated co-exposure of residential locations to air and traffic noise, with potential to impact on stressors relevant to resident’s health. Traffic noise levels are significant and reach limits which during peak hours, expose buildings near crossings to noise levels exceeding 80 dB(A). Practical efforts can be made to lower noise levels and air concentrations at sources and use strategies to protect the urban population from noise and air pollutant exposure [4,25].

Controlling and changing the traffic regime are two measures that can use to reduce noise. This approach can be achieved by enforcing speed limits, enhancing pedestrian zones, and establishing “ecological traffic lights” with driver notifications such as “Please turn off the engine”. 

Since road traffic is the primary noise source, monitoring the amount of noise created by each vehicle is critical. Vehicles are now built to a standard that complies with the maximum levels of noise emissions allowed by law. However, there is an issue with outdated automobiles still on the roads, and more robust limitations on vehicle technical examinations are required. It is also feasible to impose extra levies on noisier vehicles at the local level or to prohibit vehicles with excessive noise from driving.

In addition to the solutions, installing protective sound barriers along congested highways and zoning with settlement greening can be implemented, which, in addition to improving the microclimate.

The application of GIS to develop one framework to investigate the joint association of environmental noise and air pollutants provides opportunity to consider one of these two linked exposures run the risk of overstating the impact because of the studied exposure while understating the impact of all two environmental exposures taken together.

Careful consideration of the spatial unit of analysis is necessary, and the inclusion of within-unit distribution of correlations within statistical models should be considered where this information is available.

## Figures and Tables

**Table 1:** Characteristics of analysed road.

	EDB	GT	GK	EDK	GHS
Geographical characteristics	The terrain of Approach 1 is flat, while approaches 2, 3, and 4 are falling or rising depending on the direction of movement	The terrain of approaches 1 and 3 is flat, while approaches 2 and 4 are falling or rising, depending on the direction of movement.	The terrain on approaches 1, 4, 2 is flat, while approaching 3 is under a slope, i.e., an ascent depending on the movement’s direction.	The terrain of approaches 1 and 3 is flat, while approaches 2 and 4 are falling or rising, depending on the direction of movement.	The terrain on approaches 1, 4, 2 is flat, while approaching 3 is under a slope, i.e., an ascent depending on the movement’s direction.
Characteristics of the space/ objects in the analysed area	Residential buildings, catering facilities, business facilities, public buildings, etc	Residential buildings, catering facilities, business facilities, educational institutions, etc.	Residential buildings, catering facilities, business facilities, educational institutions, etc.	Residential buildings, catering facilities, business facilities, etc	Residential buildings, catering facilities, business facilities, educational institutions, etc.
Characteristics of the road	Two-way traffic takes place on the approaches to all traffic routes. Approaches 1, 2, and 4 have three lanes, while approach 3 has two lanes per road. The main surface of the road is asphalt. Vertical traffic lights regulate traffic at the intersection.	Two-way traffic takes place on the approaches to all roads. The number of lanes is four for directions 1, 2, and 4, while direction 3 has three lanes. The main surface of the road is asphalt. Vertical traffic lights regulate traffic at the intersection.	Two-way traffic takes place on the approaches to all roads. The number of lanes is four for directions 1, 2, and 4, while direction 3 has three lanes. The main surface of the road is asphalt. Vertical traffic lights regulate traffic at the intersection.	Two-way traffic takes place on the approaches to all roads. The number of lanes is four for roads 1 and 2, while directions 3 and 4 have three lanes. The main surface of the road is asphalt. Vertical traffic lights regulate traffic at the intersection.	Two-way traffic takes place on the approaches to all traffic routes. Approaches 1, 2, and 4 have three lanes, while approach 3 has two lanes per road. The main surface of the road is asphalt. Vertical traffic lights regulate traffic at the intersection

**Table 2:** Spearman correlations between frequency components of road noise levels, air pollutants, and traffic flow rates.

Type of Vehicle	Annual Daily Traffic Flow	31.5 Hz	Low 63 Hz	Freq 125 Hz	250 Hz	Mid 500 Hz	Freq 1 KHz	2 KHz	High 4 KHz	Freq 8 KHz	NO_2_ µg/m^3^	PM_10_ µg/m^3^	PM_2.5_ µg/m^3^
two wheels motors	235	0.000	0.600	0.600	−0.103	0.410	0.335	0.400	0.400	0.700	0.300	0.447	0.224
Car and taxi	4468	0.000	0.900 *	0.900 *	−0.205	0.821	0.671	0.500	0.500	0.800	0.300	0.224	0.447
buses	935	−0.707	0.000	0.600	0.205	0.718	0.783	0.600	0.600	0.500	−0.700	−0.894 *	−0.447
Igvs	7158	−0.354	0.500	0.900 *	−0.205	0.821	0.894 *	0.900 *	0.900 *	1.000 **	−0.300	−0.224	−0.112
All hgvs	1064	−0.707	0.200	0.800	0.205	0.718	0.783	0.800	0.800	0.900 *	−0.500	−0.447	−0.447

lgvs-low goods vehicles hgvs-heavy goods vehicles, * Correlation is significant at the 0.05 level (2-tailed), ** Correlation is significant at the 0.01 level (2-tailed).

**Table 3:** Relationship between joint annual average road traffic noise frequency component and concentration of NO_2_, PM_10_ and PM_2.5_ in all the five monitoring sites.

		NO_2_	PM_10_	PM_2.5_	LOW	MID	HIGH
NO_2_	Spearman’s rho	-					
	*p*-value	-					
PM_10_	Spearman’s rho	0.380 ***	-				
	*p*-value	<0.001	-				
PM_2.5_	Spearman’s rho	0.345 ***	0.897 ***	-			
	*p*-value	<0.001	<0.001	-			
LOW	Spearman’s rho	−0.083	0.042	0.098	-		
	*p*-value	0.114	0.517	0.127	-		
MID	Spearman’s rho	−0.012	0.081	0.145 *	0.852 ***	-	
	*p*-value	0.823	0.208	0.024	<0.001	-	
HIGH	Spearman’s rho	−0.054	0.063	0.119	0.864 ***	0.976 ***	-
	*p*-value	0.308	0.328	0.063	<0.001	<0.001	-

Note. * *p* < 0.05, *** *p* < 0.001.

## Data Availability

A dataset will be made available upon request to the corresponding authors one year after the publication of this study. The request must include a statistical analysis plan.
